# IL4I1-catalyzed tryptophan metabolites mediate the anti-inflammatory function of cytokine-primed human muscle stem cells

**DOI:** 10.1038/s41420-023-01568-x

**Published:** 2023-07-28

**Authors:** Muqiu Zuo, Jiankai Fang, Peiqing Huang, Shisong Liu, Pengbo Hou, Shiqing Wang, Zhanhong Liu, Chao Feng, Lijuan Cao, Peishan Li, Yufang Shi, Changshun Shao

**Affiliations:** 1 The Third Affiliated Hospital of Soochow University, Institutes for Translational Medicine, State Key Laboratory of Radiation Medicine and Protection, Suzhou Medical College of Soochow University, Suzhou, Jiangsu 215123 China; 2 Department of Experimental Medicine and Biochemical Sciences, TOR, University of Rome “Tor Vergata”, Rome, 00133 Italy; 3 Shanghai Institute of Nutrition and Health, Chinese Academy of Sciences, Shanghai, 200025 China

**Keywords:** Immunosuppression, Muscle stem cells

## Abstract

Muscle stem cells (MuSCs) have been demonstrated to exert impressive therapeutic efficacy in disease settings through orchestrating inflammatory microenvironments. Nevertheless, the mechanisms underlying the immunoregulatory property of MuSCs remain largely uncharacterized. Here, we showed that interleukin-4-induced-1 (IL4I1), an essential enzyme that catalyzes indole metabolism in humans, was highly expressed in human MuSCs exposed to IFN-γ and TNF-α. Functionally, the MuSCs were found to inhibit the infiltration of neutrophils into sites of inflammation in a IL4I1-dependent manner and thus ameliorate acute lung injury in mice. Mechanistically, the indole metabolites, including indole-3-pyruvic acid (I3P) and indole-3-aldehyde (I3A), produced by IL4I1, acted as ligands to activate aryl hydrocarbon receptor (AHR), leading to augmented expression of TNF-stimulated gene 6 (TSG-6) in inflammatory cytokine-primed MuSCs. Furthermore, I3P administration alone suppressed neutrophil infiltration into damaged lungs. I3P could also reduce the level of reactive oxygen species in neutrophils. Therefore, our study has uncovered a novel mechanism by which MuSCs acquire their immunoregulatory property and may help to develop or optimize MuSC-based therapies for inflammatory diseases.

## Introduction

Muscle stem cells (MuSCs), also known as satellite cells, reside in an unique niche between the muscle sarcolemma and the basal lamina of individual myofibers, and contribute to the maintenance and repair of tissue architectural structures through both self-renewal and myogenic differentiation [1, 2]. While MuSCs are essential for muscle tissue regeneration, recent evidence has shown that they also act as critical regulators in the resolution of inflammation through releasing various paracrine factors [3]. This paracrine immunomodulatory property of MuSCs is akin to what has been well established in mesenchymal stem cells (MSCs) [4, 5]. As an example, MuSCs can act on maturing macrophages and confer them with oxidative phosphorylation-dependent anti-inflammatory properties via insulin-like growth factor-2 (IGF-2), thus ameliorating dextran sulfate sodium (DSS)-induced colitis [6]. In addition, MuSCs primed with inflammatory cytokines also reduce inflammation and promote tissue repair by producing TNF-stimulated gene 6 (TSG-6) [7]. The induction of TSG-6 by inflammatory cytokines is enhanced by tryptophan metabolites, including kynurenine (KYN) and kynurenic acid (KYNA), that are catalyzed by indoleamine 2,3-dioxygenase (IDO). IGF-2 and TSG-6 function as effectors of immunoregulation for both MuSCs and MSCs. Whether or not MuSCs can acquire their immunomodulatory property via pathways distinct from those in MSCs has not been explored.

Interleukin-4-induced-1 (IL4I1), a L-amino acid oxidase, was very recently identified as a novel potential target of cancer treatment [8]. IL4I1 promoted aryl hydrocarbon receptor (AHR)-driven malignant properties and suppressed anti-tumor immunity, even in the presence of immune checkpoint blockade (ICB) and IDO inhibition [9]. Specifically, indole-3-pyruvic acid (I3P) and indole-3-aldehyde (I3A) catalyzed by IL4I1 in tumor activated AHR signaling through receptor-ligand binding, leading to increased motility of cancer cells and diminished proliferation of cytotoxic CD8^+^ T cells [9]. Unlike arginase and IDO that function intracellularly, IL4I1, as a secretory enzyme, can modulate the functions of neighboring immune cells in the inflamed tissue microenvironment [10]. On the one hand, IL4I1 can directly reduce the stability of immune synapses between T cells and dendritic cells (DCs), thereby elevating the threshold of T cell activation and thus dampening their activation [11]. IL4I1 also regulates the polarization of macrophages, as reflected by the progressive infiltration of anti-inflammatory macrophages and the displacement of proinflammatory macrophages when IL4I1 was overexpressed [12]. In adaptive immunity, IL4I1 can suppress the proliferation of Th17 cells and promote the differentiation and activity of CD4^+^CD25^+^FOXP3^+^ regulatory T cells [13, 14], thus improving the neurological severity score and alleviating experimental autoimmune encephalomyelitis in vivo [15]. Thus, there is growing evidence that IL4I1 is a vital metabolic enzyme mediating the immunosuppressive effects in disease settings.

TSG-6, a 30-kDa secreted glycoprotein, influences intercellular 3D structures and remodels the extracellular matrix (ECM) through binding with hyaluronic acid, chondroitin sulfate and proteoglycan [16]. More importantly, it possesses a potent anti-inflammatory capacity and mediates the therapeutic effects of MSCs in an array of diseases, including myocardial infarction, acute lung injury (ALI) and psoriasis [17–19]. In one instance, TSG-6 competitively conjugates the cell-surface glycosaminoglycan (GAG) binding site of CXCL8 against heparin, thereby inhibiting the infiltration of neutrophils into sites of inflammation [20]. The extravasation of leukocytes (mainly neutrophils and monocytes) is also restrained by TSG-6 released by MSCs through binding to CD44 [21]. Furthermore, TSG-6 administration promoted the phenotypic polarization of macrophages towards an anti-inflammatory state, thereby reducing inflammation in a lipopolysaccharide (LPS)-induced ALI model [22]. Although TSG-6 can potently suppress aberrant immune responses in the inflamed tissue microenvironment, how TSG-6 expression is regulated remains to be fully characterized.

In this study, we found that MuSCs primed with IFN-γ and TNF-α exerted impressive therapeutic efficacy for LPS-induced ALI through IL4I1-mediated indole metabolism. Mechanistically, I3P and I3A produced by IL4I1 not only inhibited the infiltration of neutrophils into sites of inflammation through AHR-driven TSG-6 expression but also weakened the pathogenic phenotypes of neutrophils in the damaged lungs. Our study reveals a novel indole metabolism-dependent immunosuppressive function of MuSCs and may aid the development of MuSC-based cell therapies for inflammatory diseases.

## Results

### IL4I1 is upregulated in MuSCs primed with IFN-γ and TNF-α through NF-κB and STAT6 pathways

**Fig. 1:**
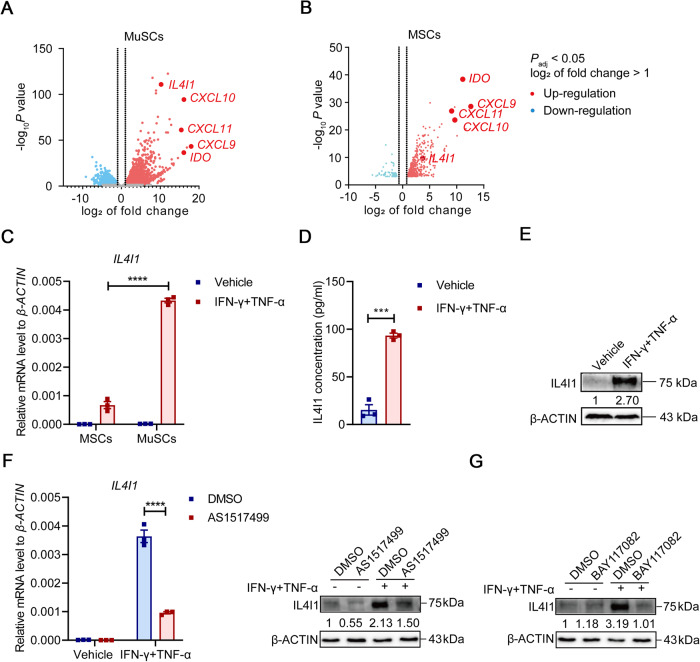
IFN-γ and TNF-α increase the expression of IL4I1 in MuSCs through NF-κB and STAT6 pathway. **A** Volcano plot of differentially expressed genes in MuSCs after the stimulation of IFN-γ and TNF-α (10 ng/ml each) for 24 h using RNA-seq. **B** Volcano plot of differentially expressed genes in MSCs after the stimulation of IFN-γ and TNF-α (10 ng/ml each) for 24 h using RNA-seq. **C** The mRNA expression of IL4I1 in MSCs and MuSCs after the stimulation of IFN-γ and TNF-α (10 ng/ml each) for 24 h was assayed by qRT-PCR. **D** The concentration of IL4I1 in the supernatants of MuSCs after the stimulation of IFN-γ and TNF-α (10 ng/ml each) for 24 h was measured by ELISA. **E** The protein expression of IL4I1 and β-ACTIN (loading control) in MuSCs after the stimulation of IFN-γ and TNF-α (10 ng/ml each) for 24 h was determined by western blotting. **F** The mRNA and protein expression levels of IL4I1 in MuSCs stimulated with IFN-γ and TNF-α (10 ng/ml each) for 24 h in the presence or absence of AS1517499 were respectively assayed by qRT-PCR and western blotting. **G** The protein expression levels of IL4I1 in MuSCs stimulated with IFN-γ and TNF-α (10 ng/ml each) for 24 h in the presence or absence of BAY117082 were assayed by western blotting. Data were shown as means ± SEM. Data were representative of three experiments with similar results. For two-group comparison, statistical analysis was performed by Student’s *t* test. ***P < 0.001; ****P < 0.0001.

Inflammatory cytokines have been demonstrated to drastically augment the expression of immunomodulatory and regenerative factors and thus boost the therapeutic efficacy of stem cells in disease settings [3, 4]. To interrogate the changes of transcriptional landscapes induced by inflammatory cytokines, we performed RNA sequencing (RNA-seq) analysis of MuSCs and MSCs stimulated by IFN-γ and TNF-α. We noted that the expression of IL4I1, which catalyzes IDO-independent tryptophan metabolism and possesses strong immunoregulatory functions, was significantly increased in both IFN-γ/TNF-α-primed MuSCs and MSCs (Fig. 1A, B). Interestingly, the induction of IL4I1 appeared to be more pronounced in MuSCs than in MSCs. This was confirmed using qRT-PCR (Fig. 1C). These results suggested that different kinds of tissue stem cells may respond to inflammatory cytokines via common mechanisms. The enhanced production of IL4I1 at protein levels in inflammatory cytokine-stimulated MuSCs was further confirmed using ELISA and western blotting analysis (Fig. 1D, E). IL4I1 expression is classically induced by IL-4 in B cells through activating STAT6 pathway [23]. Given that IFN-γ and TNF-α treatment can activate NF-κB and STAT6 pathways and drive the expression of various immunomodulatory molecules [24, 25], we employed their specific inhibitors to explore the potential regulatory mechanism of IL4I1 expression. Consistent with this idea, the expression of IL4I1 was dramatically decreased in IFN-γ/TNF-α-primed MuSCs after AS1517499 (an inhibitor of STAT6) and BAY117082 (an inhibitor of NF-κB) treatment, respectively (Fig. 1F, G). These results were consistent with the previous study showing that IL4I1 expression is increased in response to NF-κB activation and the stimulation of IL-4/STAT6 axis [26]. Taken together, these findings indicated that IFN-γ and TNF-α can induce IL4I1 expression in MuSCs through activating NF-κB and STAT6 pathways.

### IL4I1 is vital for the therapeutic effects of MuSCs on LPS-induced ALI

**Fig. 2:**
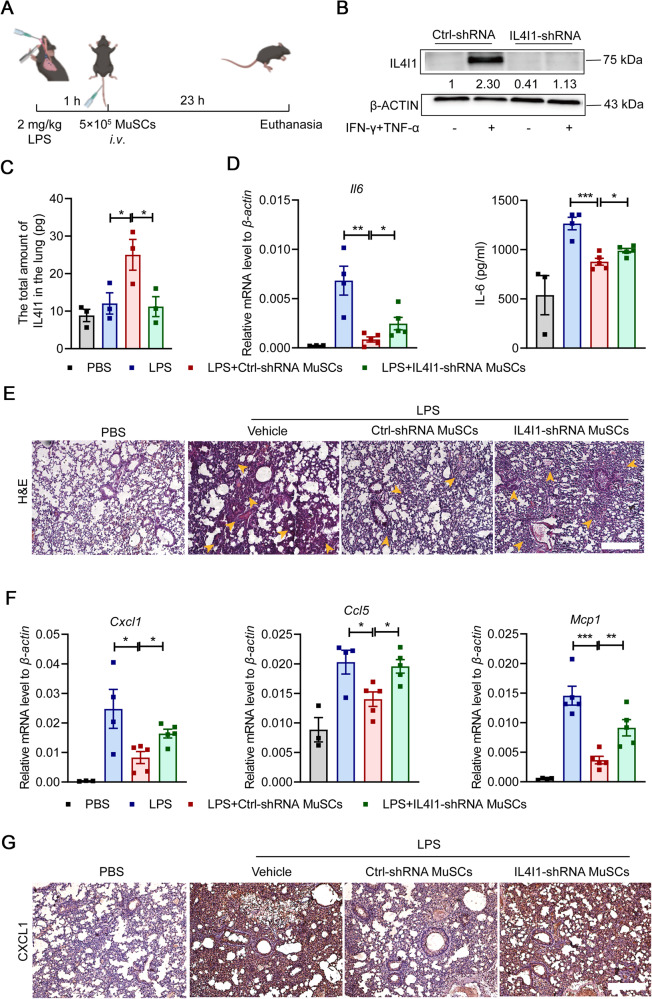
IL4I1 mediates the therapeutic effect of MuSCs on ALI. **A** The therapeutic strategy of MuSCs in the LPS-induced ALI model. Mice were treated with 2 mg/kg LPS through endotracheal infusion. 1 h later, Ctrl-shRNA MuSCs or IL4I1-shRNA MuSCs (5 × 10^5^) pretreated with IFN-γ and TNF-α (10 ng/ml each) for 24 h were intravenously injected into mice. Then, all experimental mice were euthanized after 23 h, and the lung samples were collected for further processing. **B** The efficiency of IL4I1 knockdown measured by western blotting analysis. **C** The total amount of IL4I1 protein in the lung tissue homogenates of ALI mice was determined by ELISA (PBS: *n* = 3, LPS: *n* = 3, LPS+Ctrl-shRNA MuSCs: *n* = 3, LPS + IL4I1-shRNA MuSCs: *n* = 3). **D** The expression levels of IL-6 mRNA (left) and protein (right) in the lung tissue homogenates of ALI mice were respectively determined by qRT-PCR and ELISA. **E** Lung tissues of mice with various treatments were fixed for H&E staining. Yellow arrowhead, area of widespread septal thickening with increased air-space cellularity and exudation and enhanced interstitial immune cell infiltration in the damaged lungs of ALI mice. Scale bars, 250 μm. **F** The expression levels of chemokines in the lung tissue homogenates of ALI mice were determined by qRT-PCR. **G** Lung tissues of mice with various treatments were stained with CXCL1 antibody. Scale bars, 250 μm (PBS: *n* = 3, LPS: *n* = 4, LPS+Ctrl-shRNA MuSCs: *n* = 5, LPS + IL4I1-shRNA MuSCs: *n* = 5). Data were shown as means ± SEM. Data were representative of three experiments with similar results. For multiple group comparison, statistical analysis was performed by one-way ANOVA test. *P < 0.05; **P < 0.01; ***P < 0.001.

To further investigate the immunoregulatory function of IL4I1 in IFN-γ/TNF-α-primed MuSCs, we established stable IL4I1 knockdown cell line (IL4I1-shRNA MuSCs) using lentivirus transfection and injected intravenously IL4I1-shRNA MuSCs into mice suffering from LPS-induced ALI (Fig. 2A, B). There was increased IL4I1 level in the lungs of ALI mice when Ctrl-shRNA MuSCs, but not IL4I1-shRNA MuSCs, were applied, suggesting that secreted IL4I1 was primarily derived from infused MuSCs (Fig. 2C). Consistent with this, the inhibitory effect on IL-6 levels, an important indicator for the progression of ALI [27], was slightly reduced with IL4I1-shRNA MuSC administration (Fig. 2D). Furthermore, hematoxylin and eosin (H&E) staining of lung tissue sections revealed widespread septal thickening, significantly increased air-space cellularity and exudation, and enhanced interstitial immune cell infiltration in the damaged lungs of mice treated with LPS. Importantly, administration of Ctrl-shRNA MuSCs resulted in significant amelioration. In contrast, the therapeutic effect of IL4I1-shRNA MuSCs was noticeably compromised (Fig. 2E). Additionally, the expression of chemokines, including *Cxcl1*, *Ccl5*, and *Mcp1*, responsible for the recruitment of inflammatory cells, was markedly reduced in lung tissues after Ctrl-shRNA MuSC infusion. IL4I1 knockdown abolished the suppressive effects of MuSCs on the expression of these chemokines (Fig. 2F). We next performed immunohistochemical staining of CXCL1 that mediates the infiltration of neutrophils. As expected, IL4I1-shRNA MuSC administration failed to decrease CXCL1 expression in the damaged lungs of mice suffering ALI (Fig. 2G). Taken together, these data demonstrated that the beneficial effects of MuSCs in ALI mice depend on IL4I1.

### IL4I1 decreases the infiltration of neutrophils in lung tissues

**Fig. 3:**
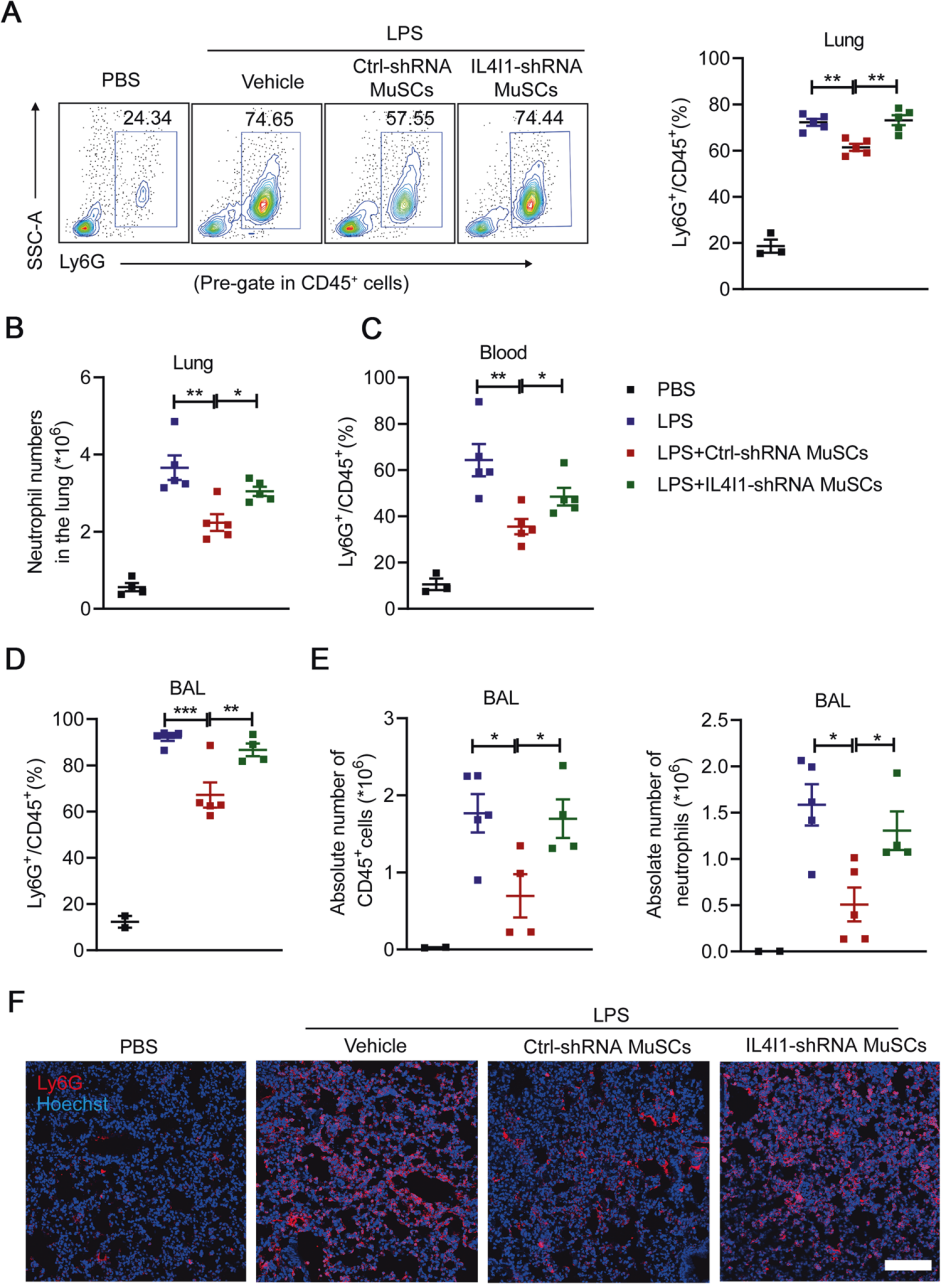
Secreted IL4I1 from MuSCs suppresses neutrophil infiltration into lung tissues in ALI mice. **A**, **B** Mice were treated with 2 mg/kg LPS through endotracheal infusion. 1 h later, Ctrl-shRNA MuSCs or IL4I1-shRNA MuSCs (5 × 10^5^) pretreated with IFN-γ and TNF-α (10 ng/ml each) for 24 h were intravenously injected into mice. The proportion and absolute numbers of neutrophils in the left lung tissues of ALI mice were determined by flow cytometry analysis. **C** The proportion of neutrophils in the blood of ALI mice was measured by flow cytometry analysis (PBS: *n* = 3, LPS: *n* = 5, LPS+Ctrl-shRNA MuSCs: *n* = 5, LPS + IL4I1-shRNA MuSCs: *n* = 5). **D** The proportion of neutrophils in the BAL fluid of ALI mice was measured by flow cytometry analysis. **E** The absolute numbers of total immune cells including neutrophils in the BAL fluid of ALI mice were determined by flow cytometry analysis (PBS: *n* = 2, LPS: *n* = 5, LPS+Ctrl-shRNA MuSCs: *n* = 5, LPS + IL4I1-shRNA MuSCs: *n* = 4). **F** The neutrophil infiltration into lung tissues was evaluated by immunofluorescence staining of Ly6G (red) and Hoechst (blue). Scale bars, 120 μm. Data were shown as means ± SEM. Data were representative of three experiments with similar results. For multiple group comparison, statistical analysis was performed by one-way ANOVA test. *P < 0.05; **P < 0.01; ***P < 0.001.

Increased infiltration of neutrophils characterizes the development and progression of ALI, and excessive and prolonged activation of neutrophils can lead to the destruction of basement membranes, thereby increasing permeability of the alveolar capillary barrier in lungs [28]. To further determine the role of IL4I1 in regulating the dynamics of inflammatory cells in ALI mice, we performed flow cytometry analysis of immune cell populations in lungs, blood and bronchoalveolar lavage (BAL) fluid (Fig. S2). As expected, Ctrl-shRNA MuSC administration significantly diminished the proportion and number of infiltrated neutrophils in lung tissues (Fig. 3A, B). Furthermore, the proportion of circulating neutrophils in total immune cell populations was obviously decreased in the blood of ALI mice (Fig. 3C), suggesting that MuSCs can exert their therapeutic effects in a systemic manner. In addition, infusion of Ctrl-shRNA MuSCs reduced the number of immune cells, particularly neutrophils, in BAL fluid of ALI mice (Fig. 3D, E). Consistent with these results, immunofluorescence staining of Ly6G also showed decreased neutrophil infiltration in lung tissues of these mice (Fig. 3F). However, IL4I1 knockdown rendered MuSCs less effective in inhibiting the infiltration of neutrophils (Fig. 3A–F). Collectively, these results indicated that IL4I1 produced from MuSCs decreases inflammatory responses in ALI partially through inhibiting the recruitment of neutrophils into damaged lung tissues.

### IL4I1 metabolites I3P and I3A promote the production of TSG-6 in MuSCs

**Fig. 4:**
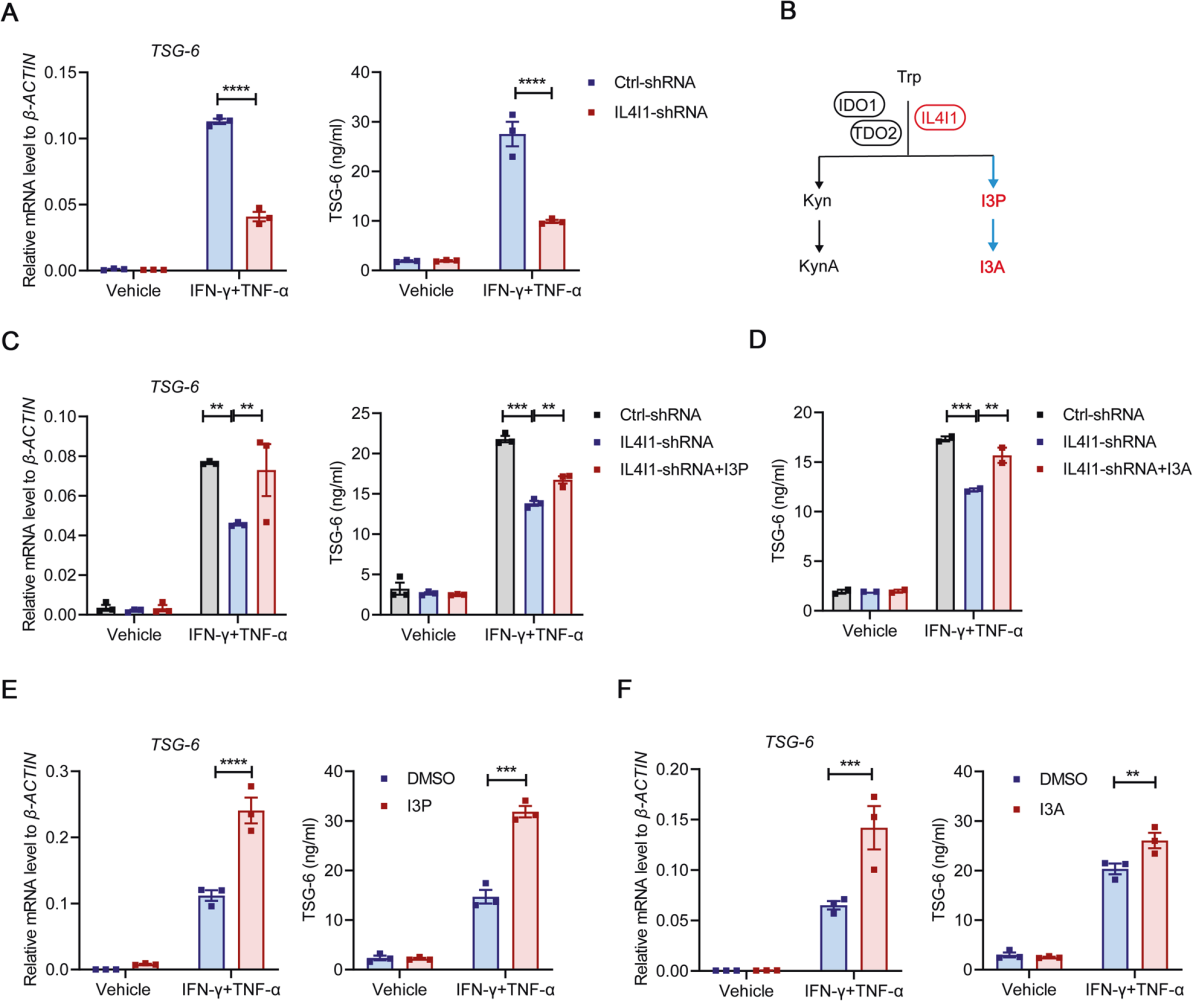
I3P and I3A promote TSG-6 production in MuSCs. **A** The mRNA and protein levels of TSG-6 in Ctrl-shRNA MuSCs and IL4I1-shRNA MuSCs stimulated with IFN-γ and TNF-α (10 ng/ml each) for 24 h were respectively measured by qRT-PCR and ELISA. **B** A schematic diagram illustrating tryptophan metabolism in humans via different catabolic routes. **C** The mRNA and protein levels of TSG-6 in Ctrl-shRNA MuSCs and IL4I1-shRNA MuSCs stimulated with IFN-γ and TNF-α (10 ng/ml each) for 24 h in the presence or absence of I3P (50 μM) were respectively measured by qRT-PCR and ELISA. **D** The concentration of TSG-6 in the supernatants of Ctrl-shRNA MuSCs and IL4I1-shRNA MuSCs stimulated with IFN-γ and TNF-α (10 ng/ml each) for 24 h in the presence or absence of I3A (100 μM) was determined by ELISA. **E** The mRNA and protein levels of TSG-6 in MuSCs stimulated with IFN-γ and TNF-α (10 ng/ml each) for 24 h in the presence or absence of I3P (50 μM) were respectively measured by qRT-PCR and ELISA. **F** The mRNA and protein levels of TSG-6 in MuSCs stimulated with IFN-γ and TNF-α (10 ng/ml each) for 24 h in the presence or absence of I3A (100 μM) were respectively measured by qRT-PCR and ELISA. Data were shown as means ± SEM. Data were representative of three experiments with similar results. For two-group comparison, statistical analysis was performed by Student’s *t* test. For multiple group comparison, statistical analysis was performed by one-way ANOVA test. **P < 0.01; ***P < 0.001; ****P < 0.0001.

TSG-6 possesses strong tissue-protective and anti-inflammatory properties in ALI through various mechanisms of action [29–32]. Our previous studies also found that inflammatory cytokine-primed MSCs and MuSCs can respectively alleviate LPS-induced ALI and DSS-induced colitis through TSG-6 production mediated by IDO-driven tryptophan metabolism [7, 18]. Paralleling to IDO, IL4I1-catalyzed indole metabolism is another important approach of tryptophan catabolism in mice and humans. Thus, we further determined whether IL4I1 could regulate the expression of TSG-6 in MuSCs through this modality of action. IL4I1 knockdown was found to dramatically reduce TSG-6 mRNA and protein levels in IFN-γ/TNF-α-primed MuSCs (Fig. 4A). As a L-amino acid oxidase independent of KYN pathway initiated by IDO1/tryptophan-2,3-dioxgenase 2 (TDO2), IL4I1 converts tryptophan into indole metabolites I3P and I3A (Fig. 4B), which exhibit powerful anti-inflammatory functions during the development and progression of various tumors [9]. To explore whether IL4I1 could promote the expression of TSG-6 through its downstream metabolites I3P and I3A, we conducted a rescue experiment in IL4I1-shRNA MuSCs with indole metabolites. Supplementation with I3P or I3A indeed restored TSG-6 expression in IFN-γ/TNF-α-primed MuSCs with IL4I1 knockdown (Fig. 4C, D). While TSG-6 expression was greatly induced in MuSCs stimulated with inflammatory cytokines, exogenous I3P and I3A could further increase TSG-6 production in these MuSCs (Fig. 4E, F). Together, these data suggested that IL4I1 upregulates TSG-6 expression through indole metabolites I3P and I3A in inflammatory cytokine-primed MuSCs.

### I3P and I3A promote TSG-6 production in MuSCs via AHR signaling

**Fig. 5:**
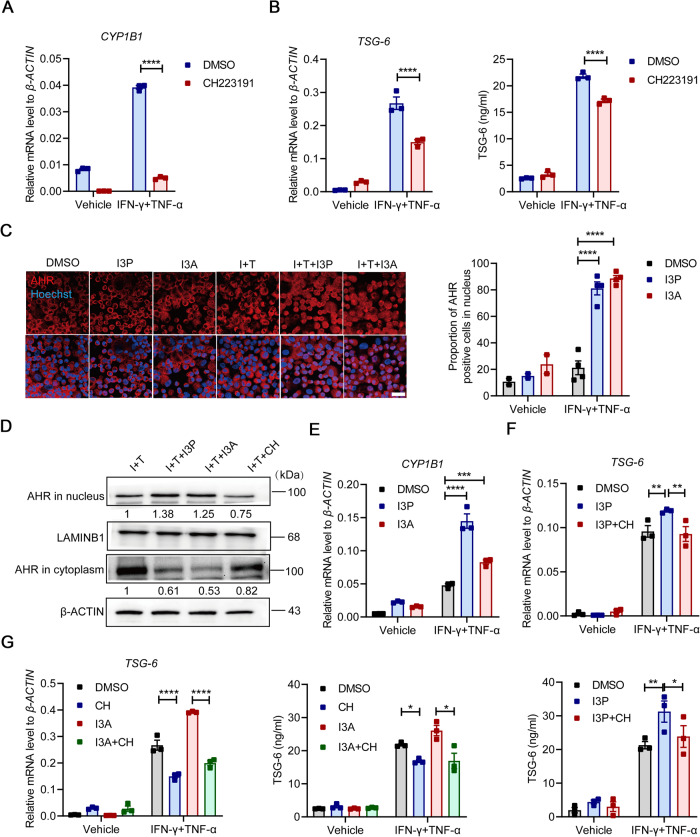
I3P and I3A activate AHR signaling pathway to enhance TSG-6 production in MuSCs. **A** The *CYP1B1* expression in MuSCs stimulated with IFN-γ and TNF-α (10 ng/ml each) in the presence or absence of CH223191 (100 μM) for 24 h was measured by qRT-PCR. **B** The mRNA and protein levels of TSG-6 in MuSCs stimulated with IFN-γ and TNF-α (10 ng/ml each) in the presence or absence of CH223191 (100 μM) for 24 h were respectively measured by qRT-PCR and ELISA. **C** Immunofluorescence staining of AHR in MuSCs treated with IFN-γ (I, 10 ng/ml) and TNF-α (T, 10 ng/ml) in the presence or absence of I3P (50 μM) and I3A (100 μM) for 24 h. Scale bars, 40 μm. **D** MuSCs treated with IFN-γ (I, 10 ng/ml) and TNF-α (T, 10 ng/ml) in the presence or absence of I3P (50 μM), I3A (100 μM) and CH223191 (CH, 100 μM) were subjected to the cytoplasmic and nuclear extraction, and the distribution of AHR in cytoplasm and nucleus was analyzed by western blotting analysis. β-ACTIN and LAMINIB1 were served as loading controls for cytoplasmic and nuclear proteins, respectively. **E** The *CYP1B1* expression in MuSCs stimulated with IFN-γ and TNF-α (10 ng/ml each) in the presence or absence of I3P (50 μM) and I3A (100 μM) for 24 h was measured by qRT-PCR. **F** The mRNA and protein levels of TSG-6 in MuSCs stimulated with IFN-γ and TNF-α (10 ng/ml each) in the presence or absence of I3P (50 μM) and CH223191 (CH, 100 μM) for 24 h were respectively measured by qRT-PCR and ELISA. **G** The mRNA and protein levels of TSG-6 in MuSCs stimulated with IFN-γ and TNF-α (10 ng/ml each) in the presence or absence of I3A (100 μM) and CH223191 (CH, 100 μM) for 24 h were respectively measured by qRT-PCR and ELISA. Data were shown as means ± SEM. Data were representative of three experiments with similar results. For two-group comparison, statistical analysis was performed by Student’s *t* test. For multiple group comparison, statistical analysis was performed by one-way ANOVA test. *P < 0.05; **P < 0.01; ***P < 0.001; ****P < 0.0001.

We previously found that AHR is highly expressed in inflammatory cytokine-primed MuSCs [7]. It is one of the most recognized members of the basic helix-loop-helix-per-arnt-Sim (bHLH-PAS) transcription factor superfamily. When translocated to the nucleus after binding to ligands, AHR will form heterodimers with the AHR nuclear translocation (ARNT), which can initiate the transcription machinery of multiple genes involved in various cellular processes through binding to distinct biological response element (XRE) sequences [33]. TSG-6, in particular, is a target gene of AHR [18]. Thus, we next attempted to determine whether I3P and I3A could promote TSG-6 expression in MuSCs through AHR signaling. When CH223191 was added to antagonize the nuclear translocation of AHR, the induction of *CYP1B1*, a classical target gene of AHR signaling, was indeed blocked in IFN-γ/TNF-α-primed MuSCs (Fig. 5A). Consistent with this, AHR blockade led to diminished TSG-6 expression in MuSCs (Fig. 5B). Furthermore, I3P and I3A significantly increased the accumulation of AHR in the nucleus, indicating that I3P and I3A could activate AHR signaling in activated MuSCs (Fig. 5C, D). The enhanced AHR activity was further supported by the increased expression of *CYP1B1* by I3P and I3A administration (Fig. 5E). However, AHR blocking through CH223191 abolished the enhancement of TSG-6 expression caused by I3P and I3A in IFN-γ/TNF-α-primed MuSCs (Fig. 5F, G). Collectively, these results demonstrated that I3P and I3A promote the expression of TSG-6 in MuSCs through activating AHR.

### I3P administration alleviates ALI

**Fig. 6:**
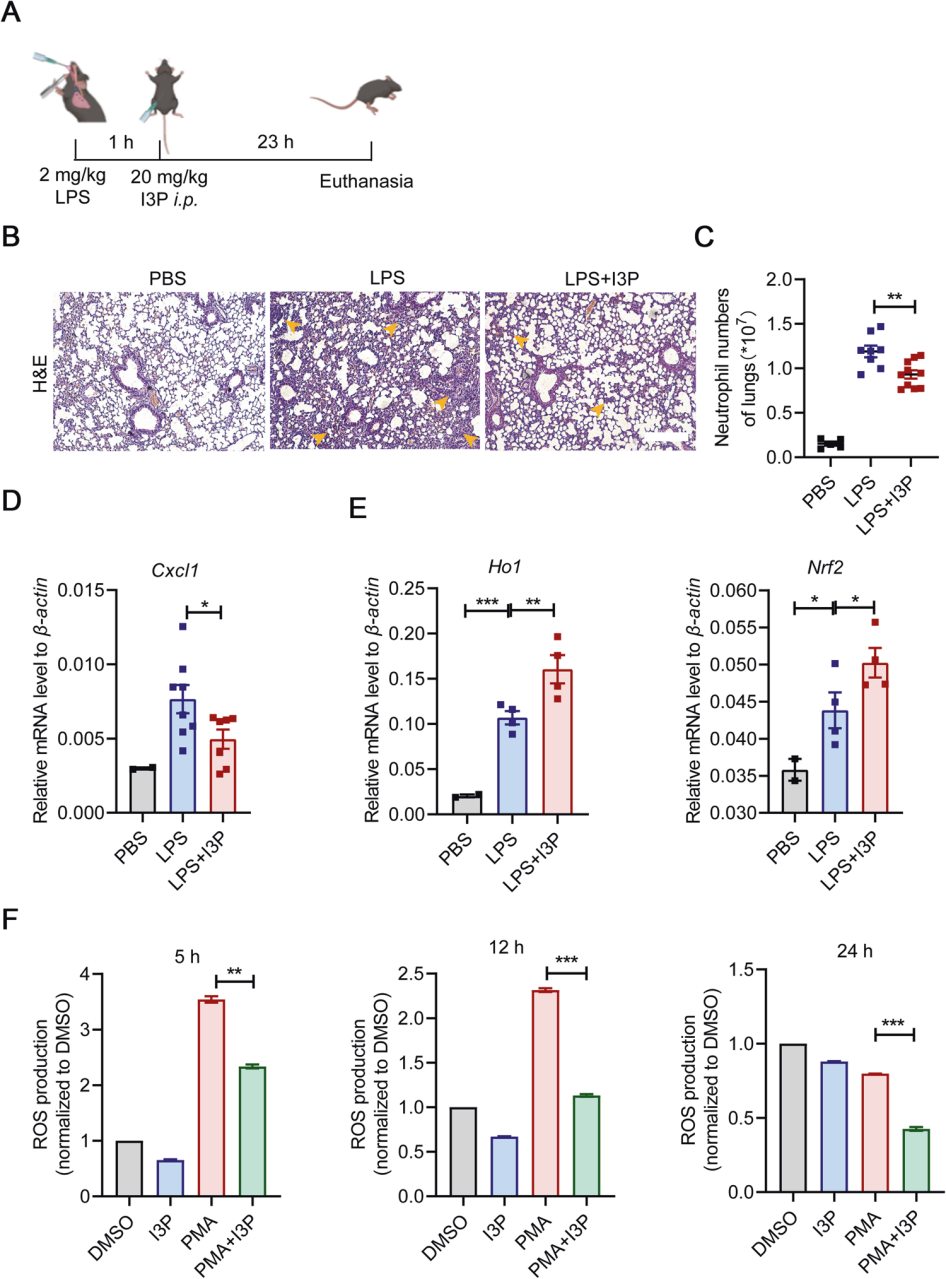
I3P administration suppresses neutrophil infiltration and enhances oxidation resistance in damaged lungs. **A** The therapeutic strategy of I3P in the LPS-induced ALI model. Mice were treated with 2 mg/kg LPS through endotracheal infusion. 1 h later, I3P (20 mg/kg) were intraperitoneally injected into mice. Then, all experimental mice were euthanized after 23 h, and the lung samples were collected for further processing. **B** Lung tissues of mice with various treatments were fixed for H&E staining. Yellow arrowhead, area of widespread septal thickening with increased air-space cellularity and exudation and enhanced interstitial immune cell infiltration in the damaged lungs of ALI mice. Scale bars, 250 μm. **C** The number of neutrophils in the lungs of ALI mice was measured by flow cytometry analysis (PBS: *n* = 5, LPS: *n* = 8, LPS + I3P: *n* = 10). **D** The expression levels of *Cxcl1* in the lung tissue homogenates of ALI mice were determined by qRT-PCR (PBS: *n* = 2, LPS: *n* = 8, LPS + I3P: *n* = 7). **E** The expression levels of *Ho1* and *Nrf2* in neutrophils infiltrating into the lungs were determined by qRT-PCR (PBS: *n* = 2, LPS: *n* = 4, LPS + I3P: *n* = 4). **F** ROS levels in PMA-activated neutrophils treated with I3P (200 μM) were measured by flow cytometry analysis. Data were shown as means ± SEM. Data were representative of three experiments with similar results. For multiple group comparison, statistical analysis was performed by one-way ANOVA test. *P < 0.05; **P < 0.01; ***P < 0.001.

To further investigate the therapeutic potential of I3P in disease settings, we intraperitoneally injected I3P into mice suffering from LPS-induced ALI (Fig. 6A). Consistent with MuSC therapy, I3P administration resulted in significant amelioration, as reflected by reduced septal thickening, significant decrease in air-space cellularity and exudation, and diminished interstitial immune cell infiltration in ALI mice (Fig. 6B). Flow cytometry analysis also showed that the number of infiltrating neutrophils in the damaged lungs was obviously reduced in ALI mice treated with I3P (Fig. 6C). In addition, there was decreased expression of *Cxcl1* in mice treated with I3P (Fig. 6D). While reactive oxygen species (ROS) production is essential for neutrophils to combat bacteria and other invading pathogens, excessive ROS can induce tissue damage and exacerbate pulmonary inflammation [34–36]. Given the recent report that I3P can activate an anti-oxidative gene expression program [37], we speculated that I3P might also directly act on neutrophils and reduce their tissue damaging activity mediated by ROS. Indeed, I3P administration induced the expression of anti-oxidative genes heme oxygenase 1 (*Ho1*) and nuclear factor erythroid 2-related factor 2 (*Nfe2l2*, also known as *Nrf2*) in infiltrating neutrophils in lungs (Fig. 6E). Consistent with this, ROS level in neutrophils treated with phorbol myristate acetate (PMA) was reduced by concomitant I3P addition (Fig. 6F), suggesting that the alleviation of disease symptoms in ALI mice treated with I3P could also be attributed to impaired pathogenic phenotypes of neutrophils in damaged lungs. Collectively, these data suggested that I3P administration can inhibit the infiltration of neutrophils and protect the lungs against ROS-mediated oxidative damage from neutrophils, thereby exerting the therapeutic efficacy for ALI.

## Discussion

Tissue stem cells are generally known for their ability to differentiate into various types of daughter cells that participate in organogenesis, maintain tissue integrity or carry out other physiological functions. The premise of stem cell-based therapy is largely based on the assumption that tissue stem cells may replace the missing or damaged functional cells in the injured organ. However, only few transplanted stem cells are capable of differentiating into the desired cells to complete local physical reconstitution through their ‘stemness’ [38]. Emerging evidence suggests that the therapeutic effects of stem cells may rely on their ability in enabling damaged or dysfunctional tissues to form a balanced inflammatory and regenerative microenvironment, a paradigm also known as ‘empowerment’ [4, 39, 40]. A recent study found that the beneficial therapeutic effect of cardiac stem cells (CSCs) was attributed to CSC-mediated immunomodulation for CCR2^+^ and CX3CR1^+^ macrophages rather the production of new cardiomyocytes through lineage differentiation. These educated macrophages helped to decrease cardiac fibroblast activities and promote remodeling of ECM, thereby halting progressive heart fibrosis [41]. We recently showed that MuSCs harbor potent immunoregulatory properties in disease settings [6]. Importantly, these abilities can be further strengthened through preconditioning with inflammatory cytokines. Mechanistically, priming with IFN-γ and TNF-α enhanced TSG-6 production through IDO-mediated tryptophan catabolism along the KYN pathway, thereby endowing MuSCs with more powerful therapeutic potency in DSS-induced colitis [7]. These results demonstrated that the immunosuppressive properties of MuSCs are governed by tryptophan metabolism.

The KYN pathway initiated by IDO1 or TDO2 utilizes tryptophan to produce KYNA and other bioactive molecules that function as endogenous AHR ligands [42, 43]. High expression of IDO in inflammatory cytokine-primed stem cells is essential for their therapeutic effects in disease settings [3, 4]. Therein, KYNA activates AHR signaling, and thus upregulates the transcriptional expression of TSG-6 in IFN-γ/TNF-α-activated human MSCs and MuSCs [7, 18]. Interestingly, IL4I1-catalyzed indole generation is recently emerging as an alternate route of tryptophan catabolism in humans. Indole metabolites, including I3P and I3A, also function as potent AHR agonists. This route enables cancer cells to circumvent the anti-tumor immunity unleashed by IDO inhibitors in cancer therapy [9]. It also suggests that indole metabolites and their effects on AHR activity are not solely attributed to microbial metabolism. We found that IL4I1 is upregulated to a greater extent than IDO in inflammatory cytokine-primed human MuSCs, suggesting that whereas there are two routes along which tryptophan is metabolized to generate bioactive small molecules that modulate immune functions, the preferred route may vary depending on cell types. In this context, TSG-6 production through IDO-initiated KYN pathway may be partially responsible for the therapeutic efficacy of MuSCs for ALI.

In addition to activating AHR to promote the expression of TSG-6 in MuSCs, extracellular I3P catalyzed by IL4I1 can also influence the functions of neighboring immune cells. Actually, I3P administration could directly curb disease symptoms of ALI mice through distinct mechanisms of action. Specifically, there was decreased infiltration of neutrophils into damaged lung tissues of ALI mice after I3P treatment, in which I3P inhibited CXCL1-mediated chemotaxis by neutrophils towards lungs. Moreover, ROS-mediated pathogenic effects of neutrophils were significantly restrained through increased expression of anti-oxidative genes when I3P administration. Collectively, these results suggested that I3P therapy may be explored for further treatment of inflammatory diseases.

While this study reveals that the therapeutic efficacy of MuSCs for ALI is attributed to their immunosuppressive effects on neutrophils via the IL4I1-TSG6 axis, it does not rule out that IL4I1-catalyzed tryptophan metabolites may also act, directly or indirectly, on macrophages to exert their immunomodulatory effects.

**Fig. 7:**
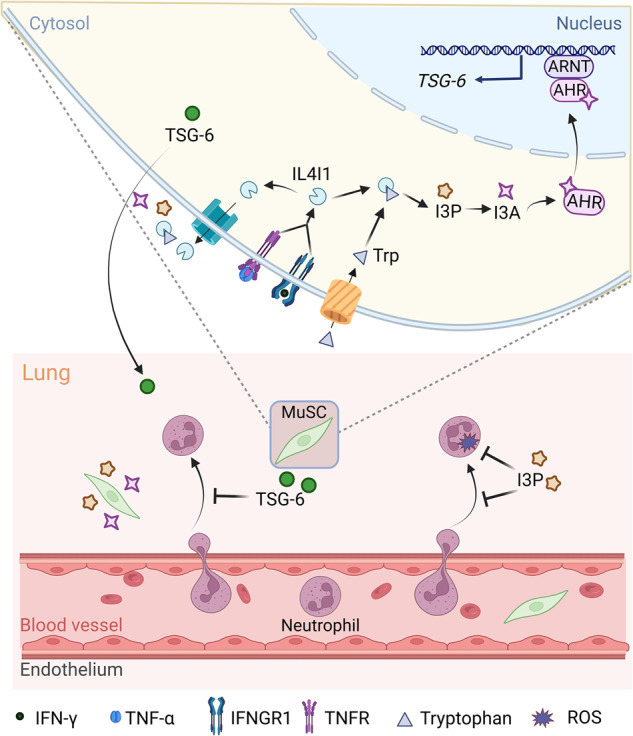
A schematic model of the anti-inflammatory effects of IL4I1 on LPS induced-ALI. IL4I1 induced by IFN-γ and TNF-α catalyzes tryptophan metabolism to produce indole metabolites I3P and I3A in MuSCs, which promote TSG-6 production through activating AHR signaling pathway, and consequently alleviate ALI. Additionally, extracellular I3P can suppress the infiltration of neutrophils into damaged lungs and reduce ROS-mediated oxidative damage from neutrophils.

In summary, we have discovered a novel mechanism by which MuSCs function as immunoregulators. When stimulated by inflammatory cytokines, MuSCs yield I3P and I3A via IL4I1-catalyzed indole metabolism. As AHR ligands, they activate AHR, thereby upregulating TSG-6 expression. The indole metabolites can also directly act on neutrophils to exert their anti-inflammatory effects (Fig. 7). These results not only highlight the importance of IL4I1 and its downstream metabolites in shaping the immunoregulatory function of MuSCs, but also provide a basis for developing indole metabolism-based new strategies for the treatment of inflammatory diseases.

## Materials and methods

### MuSC culture

MuSC culture was as previously described [7]. In brief, MuSCs were cultured in myogenic growth medium containing 1:1 mixture of DMEM Low medium and MCDB 131 medium, 20% fetal bovine serum (FBS), 1% penicillin-streptomycin (all from Gibco, MA, USA), 1% insulin-transferrin-selenium (Invitrogen, Carlsbad, CA, USA), and 10 μM p38 MAPK inhibitor (SB203580, Selleck, Houston, TX, USA), which functions to block the differentiation of MuSCs and thus maintain their self-renewal properties [44]. Cell plates and slides were pre-coated with ECM (Sigma-Aldrich, St Louis, MO, USA). All cell culture experiments were performed under 5% CO_2_ and atmospheric oxygen at 37 °C in a humidified incubator without mycoplasma contamination. All details regarding the characterization of cultured MuSCs were shown in Fig. S1.

### IL4I1 knockdown

To achieve IL4I1 knockdown, MuSCs were transfected with *IL4I1*-targeting short hairpin RNA (shRNA) carried on a lentivirus vector (PGLV3/H1/GFP/Puro, from GenePharma, Shanghai, China) and incubated with polybrene (5 μg/mL, GenePharma) for 12 h, which were named as IL4I1-shRNA MuSCs. The shRNA target sequence for *IL4I1* was 5’-GCAACTATGTGGTGGAGAAGG-3’. Those MuSCs transfected with negative control shRNA, whose target sequence was 5’-TTCTCCGAACGTGTCACGT-3’, were named as Ctrl-shRNA MuSCs. Puromycin (5 μg/mL, from Gbico) was added to the culture medium to screen successfully transfected cells.

### ALI model

Male C57BL/6J mice (6–8 weeks old) were purchased from Charles River Experimental Animal Technology Co. Ltd. (Beijing, China) and maintained under specific pathogen-free conditions of the Laboratory Animal Center of Soochow University. Animal care was in full compliance with the Guide for the Care and Use of Laboratory Animals, and the experimental protocols were approved by the Institutional Animal Care and Use Committee of Soochow University (SUDA20210916A07). The mice were housed in a 12 h light-dark cycle, and fed with irradiated food and sterile water ad libitum. All mice were acclimatized to the housing for 2 weeks before starting the experimental procedures.

For animal experiments involving the establishment of ALI model, mice were randomly grouped according to a random number table. Each group included 2 ~ 10 mice. The sample size was determined based on previous studies [18], pilot studies, the level of expected heterogeneity of samples, as well as the significance threshold (chosen at 0.05). Specifically, mice were randomly divided into PBS, LPS, LPS+Ctrl-shRNA MuSCs and LPS + IL4I1-shRNA MuSCs groups. The LPS-induced ALI model was established by an airway administration of LPS (2 mg/kg, per mouse) into mice. 1 h later, MuSCs (5 × 10^5^ cells) and I3P (20 mg/kg) were intravenously and intraperitoneally injected to mice with ALI, respectively. After 24 h LPS challenge, mice were sacrificed and BAL fluid was collected through the tracheal cannula. Blood and lung tissue samples were collected for subsequent analyses.

### RNA sequencing

Total RNA from MuSCs and MSCs was extracted using the Trizol reagents, quantified and purified through Bioanalyzer 2100 and RNA 6000 Nano LabChip Kit (Agilent, CA, USA) according to the manufacturer’s instructions. The RNA libraries were prepared and sequenced on the Illumina Novaseq™ 6000 platform by LC Bio Technology CO., Ltd (Hangzhou, China). Data analysis and visualization were performed using R packages named as ggplot2 and ggrepel (https://www.r-project.org/) and OmicStudio platform of LC Bio Technology CO., Ltd (https://www.omicstudio.cn/tool). Specifically, log processing was performed on all data obtained from RNA-seq. Log pretreatment refers to adding a value to the data before log processing, which is applicable to the case where the distribution range of data crosses orders of magnitude. In this case, log processing can enhance the visualization effect without changing the linear relationship of global data. Then, these data after log pretreatment were processed through centralization that refers to the value of a gene minus its mean across all samples. Through the above data processing, the differentially expressed genes were shown through volcano plot graphs.

### Western blotting analysis

Western blotting was performed to measure the level of IL4I1 protein in MuSCs. Total protein was extracted using RIPA lysis buffer (Beyotime, Shanghai, China). Protein concentration was determined using a BCA protein assay kit (Bio-Rad, Hercules, CA, USA). 40 μg of protein was separated by a 10% SDS-PAGE gel and transferred to a polyvinylidene fluoride membrane (Millipore, Temecula, CA, USA). After blocking with 5% BSA for 2 h, the membrane was incubated with primary antibodies overnight at 4 °C. The primary antibodies included anti-human IL4I1 antibody (Cat# MAB5684, from R&D Systems, Minneapolis, MN, USA) and anti-human β-ACTIN antibody (Cat# ab6276, from Abcam). The membrane was then washed three times with TBST and respectively incubated with horseradish peroxidase (HRP)-conjugated anti-rat second antibody (Cat# 7077, from Cell Signaling Technology, Boston, MA, USA) and anti-mouse second antibody (Cat# ab205719, from Abcam) at room temperature for 1 h. Finally, the protein levels were detected by the chemiluminescence reagent.

### qRT-PCR

Total RNA was extracted using RNAprep Pure Cell Kit (Feijie, Shanghai, China), and reverse-transcribed into cDNA with PrimeScript™ RT Master Mix (TaKaRa, Dalian, China). The levels of mRNA expression were analyzed by QuantStudio™ 6 Flex System according to the manufacturer’s instructions. The total reaction volume of 10 μl was comprised of 1 ng cDNA, 3 μl DNAase/RNAse-free water (TaKaRa), 1 μl special primers (GENEWIZ, Suzhou, Jiangsu, China), and 5 μl SYBR qPCR SuperMixplus with ROX (Novoprotein, Shanghai, China). The real-time PCR program was as follows: pre-denaturation at 95 °C for 30 s; 40 cycles of denaturation at 95 °C for 5 s; annealing and extension at 60 °C for 30 s. The total amount of mRNA was compared with endogenous *β-ACTIN* mRNA. Finally, the relative expression of mRNA was calculated using 2^–ΔΔCt^ method. Primer sequences were shown in Supplementary Table S1.

### ELISA

The supernatants of MuSCs were collected 24 h after distinct treatments to detect the concentration of TSG-6 through ELISA as previously described [7]. Briefly, a 96-well plate was pre-coated with 50 μl of 10 μg/ml anti-human TSG-6 antibody (Cat# sc-377277, from Santa Cruz, Dallas, Texas, USA) overnight, washed with PBS and blocked with the buffer containing 0.25% BSA and 0.05% Tween 20. After 1 h, the excess blocking buffer was washed away. The MuSC supernatants were added in the 96-well plate. Then, biotinylated anti-human TSG-6 antibody (Cat# BAF2104, from R&D Systems) was added and incubated for 2 h at room temperature. After washing with PBS, streptavidin-horseradish peroxidase (R&D Systems) was added for 30 min at room temperature, and developed using substrate solutions (R&D Systems).

### Immunofluorescence assay

For immunofluorescence staining of lung sections, the hydrated slides were subjected to antigen retrieval in Tris-EDTA (pH 9.0, from Yuanye, Shanghai, China) at 96 °C for 30 min. Then, lung sections were incubated with 3% bovine serum albumin (BSA, from Amresco, OH, USA) for 1 h to prevent non-specific staining before incubating with anti-mouse Ly6G antibody (Cat# ab210204, from Abcam) overnight at 4 °C. Finally, lung sections were incubated with anti-rat IgG (H + L) antibody (Alexa Fluor^®^ 594, Cat# ab150160, from Abcam) for 1 h and Hoechst 33324 (Beyotime) for 10 min at room temperature.

For cellular immunofluorescence staining, MuSCs were seeded on eight-chamber slide (Thermo Fisher) at the density of 1 × 10^4^ cells per chamber. After indicated treatment, cells were fixed with paraformaldehyde for 10 min, permeabilized with 0.5% Triton X-100 for 10 min, and blocked with 3% BSA in PBS for 1 h. The cells were then incubated with 10 μg/ml anti-human AHR antibody (Cat# ab190797, from Abcam) overnight at 4 °C. Then, cells were washed with PBS and incubated with anti-rabbit IgG (H + L) antibody (Alexa Fluor^®^ 647, Cat# A-31573, from Thermo Fisher) for 1 h at room temperature. After PBS washing, cell nucleus was stained with 1 μg/ml Hoechst 33324 (Beyotime) for 8 min at room temperature. Finally, immunofluorescence images were obtained through the Nikon ECLIPSE Ni-U microscope.

### Flow cytometry

Mice were sacrificed 24 h after LPS exposure. Lung tissues and peripheral blood mononuclear cells were collected. To obtain BAL fluid, lungs were washed three times with 1 ml PBS. The single-cell suspensions for flow cytometric analysis were pre-incubated with anti-CD16/CD32 antibody (Cat# 101301, from BioLegend, San Diego, CA, USA) to block Fc receptors, and then stained with APC anti-mouse CD45 antibody (Cat# 103137, from BioLegend) and FITC anti-mouse Ly6G antibody (Cat# 127605, from BioLegend) at 4 °C for 20 min. Then, total cell numbers of immune cells including neutrophils were determined through the Cytoflex Flow Cytometer (Beckman Coulter, CA, USA).

### Histological analysis

Isolated lung tissues were fixed in 4% paraformaldehyde, dehydrated using graded ethanol, embedded in paraffin, cut into 5-μm-thick sections, and then stained with hematoxylin and eosin (H&E). For the immunohistochemical staining of CXCL1, lung samples were stained with anti-mouse CXCL1 antibody (Cat# 12335-1-AP, from Proteintech, Wuhan, Hubei, China), followed by HRP-linked anti-rabbit secondary antibody (Maixin, Fuzhou, Fujian, China) and diaminobenzidine (Maixin). Images were obtained through the Nikon ECLIPSE Ni-U microscope. More than three fields from each mouse were selected and the images are representative for each experiment repeats.

### ROS measurement

The single cell suspension of neutrophils was stained with 20 µM dichlorofluorescin diacetate (DCFDA) and incubated for 30 min at 37 °C. Cell samples were washed with PBS. Finally, the ROS levels in neutrophils were measured through the flow cytometer after establishing forward and side scatter gates to exclude debris and cellular aggregates from analysis.

### Statistical analysis

Statistical analyses were performed using GraphPad Prism 9 (GraphPad Prism Software Inc., CA, USA). All data were presented as the mean ± SEM. For comparisons between two groups, *P* values by Student’s *t* test were reported when there was no significance in the *F*-test and in accordance with a normal distribution. For comparisons among multiple groups, one-way ANOVA followed by Tukey’s multiple comparison test was performed when no significance in the *F*-test. Each experiment was repeated three times. Notably, *P* values less than 0.05 were considered statistically significant. *P < 0.05; **P < 0.01; ***P < 0.001; ****P < 0.0001.

## Supplementary information

Supplementary figure and supplementary table

Full length uncropped original western blots

## Data Availability

Data are available from the corresponding authors upon reasonable request.
