# Supplementary material for: IL4I1-catalyzed tryptophan metabolites mediate the anti-inflammatory function of cytokine-primed human muscle stem cells
Source: Cell Death Discov. 2023 Jul 28;9:269. doi: 10.1038/s41420-023-01568-x (PMC10382538; doi:10.1038/s41420-023-01568-x)
Supplement: Supplementary file 1 — Supplementary figure and supplementary table [file 41420_2023_1568_MOESM1_ESM.docx]

**Supplementary figure**


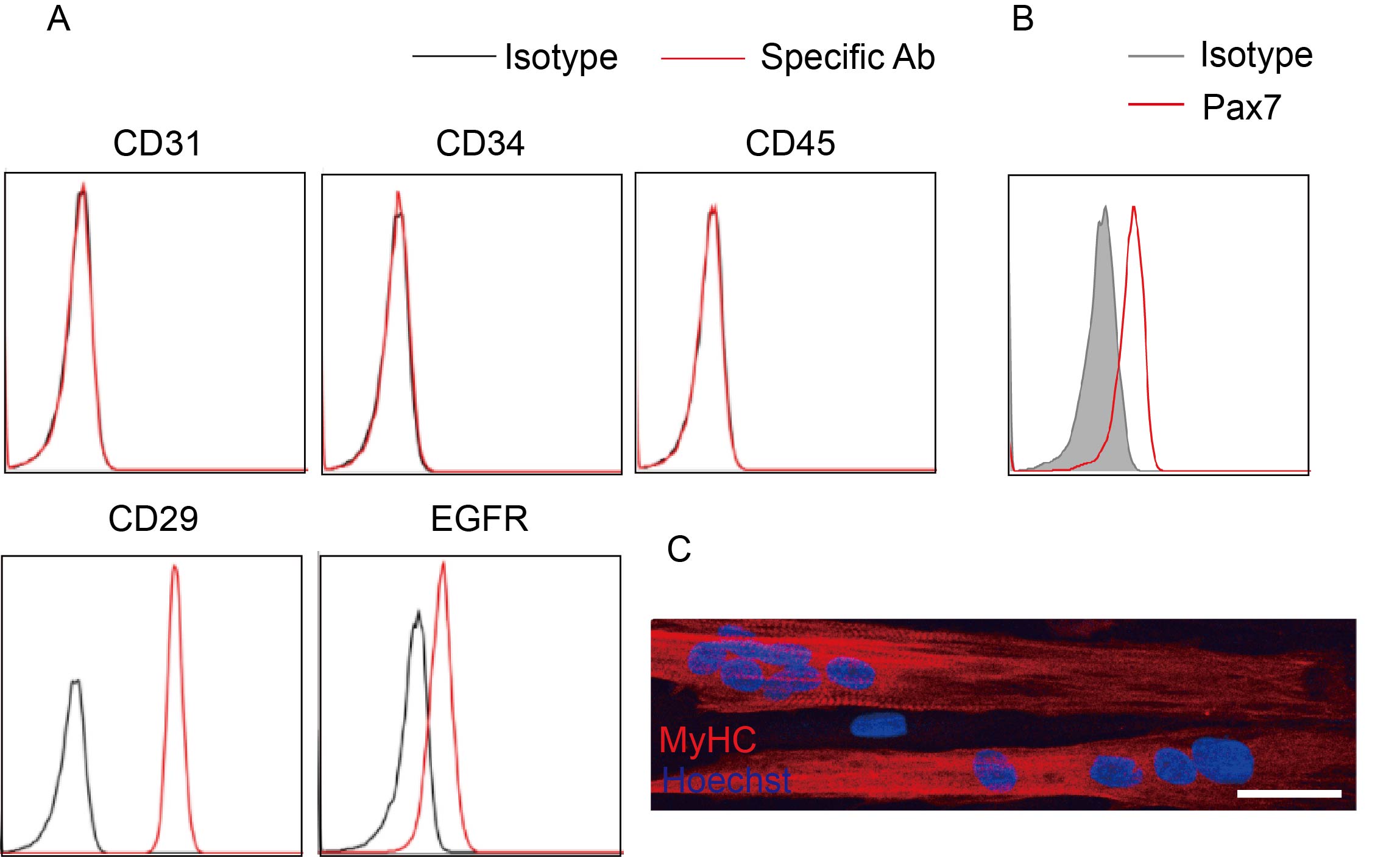


**Fig. S1 Phenotypic characterization of MuSCs and their differentiation *in vitro*.** **A** Characterization of cultured human MuSC surface markers by flow cytometric analysis. **B** Characterization of nuclear factor PAX7 in cultured human MuSCs by flow cytometric analysis. **C** Representative images of the differentiation potentials of cultured human MuSCs. Scale bar, 50 μm.


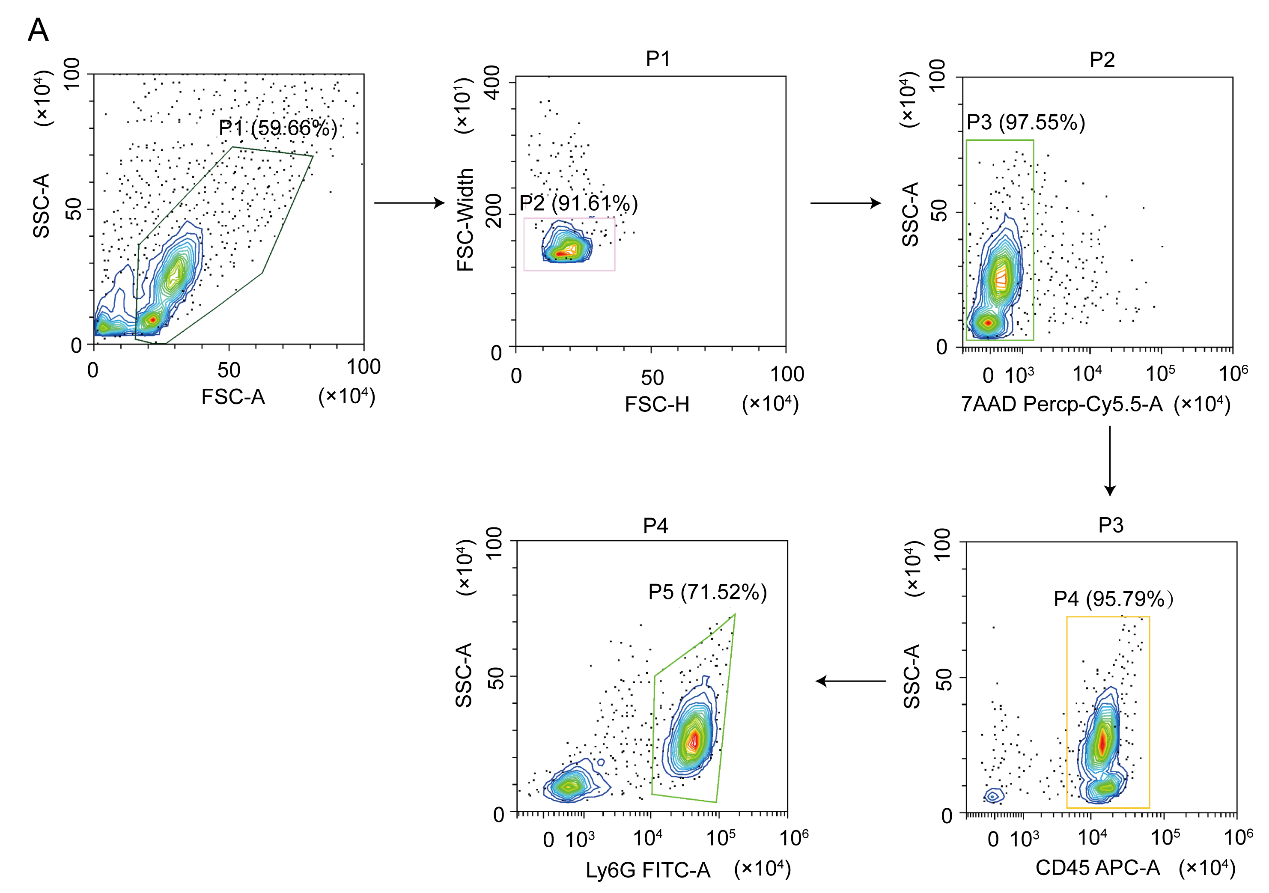


**Fig. S2 The gating strategy for determination of the different cell populations in the BAL liquid, blood and lungs**. **A** Debris and doublets were first excluded from the single cells, thereby yielding the living cell population as shown in the SSC vs. 7AAD plot. The total immune cell population was gated based on their high CD45 expression. Neutrophils were further gated based on their high Ly6G expression.

**Supplementary table**

Table S1 The primers for qRT-PCR

| Gene | Classification of primers | Sequence (5’-3’) |
| --- | --- | --- |
| *IL4I1* | Forward primer | 5’-GCCAAGACCCCTTCGAGAAAT-3’ |
|  | Reverse primer | 5’-CCGATCCTGTTATCTGCCTCC-3’ |
| *TSG-6* | Forward primer | 5’-TTTCTCTTGCTATGGGAAGACAC-3’ |
|  | Reverse primer | 5’-GAGCTTGTATTTGCCAGACCG-3’ |
| *CYP1B1* | Forward primer | 5’-GCAAGGGCATGGGAATTGAC-3’ |
|  | Reverse primer | 5’-GAGTCTCTTGGCGTCGTCAG-3’ |
| *β-ACTIN* | Forward primer | 5’-TTGCCGACAGGATGCAGAAGGA-3’ |
|  | Reverse primer | 5’-AGGTGGACAGCGAGGCCAGGAT-3’ |
| *Cxcl1* | Forward primer | 5’-CCCTCTCCTTCCTCATTCTTACA-3’ |
|  | Reverse primer | 5’-AGTCTTGAAAGCCCATGTGAAA-3’ |
| *β-actin* | Forward primer | 5’-GTGACGTTGACATCCGTAAAGA-3’ |
|  | Reverse primer | 5’-GCCGGACTCATCGTACTCC-3’ |
| *Ccl5* | Forward primer | 5’-GCTGCTTTGCCTACCTCTCC-3’ |
|  | Reverse primer | 5’-TCGAGTGACAAACACGACTGC-3’ |
| *Mcp1* | Forward primer | 5’-CTCCAAGCCAAAGTCCTTAGAG-3’ |
|  | Reverse primer | 5’-TCTCTCTTCCTCCACCACCATG-3’ |
|  | Reverse primer | 5’-GCGTTAACTGCATCTGGCTGA-3’ |
| *Nrf2* | Forward primer | 5’-GAAGCACGCTGAAGGCACAAT-3’ |
|  | Reverse primer | 5’-AGGGCCGTTCTGTTTGACAC-3’ |
| *Ho1* | Forward primer | 5’-AGGTACACATCCAAGCCGAGA-3’ |
|  | Reverse primer | 5’-CATCACCAGCTTAAAGCCTTCT-3’ |
