# Supplementary material for: IL4I1-catalyzed tryptophan metabolites mediate the anti-inflammatory function of cytokine-primed human muscle stem cells
Source: Cell Death Discov. 2023 Jul 28;9:269. doi: 10.1038/s41420-023-01568-x (PMC10382538; doi:10.1038/s41420-023-01568-x)

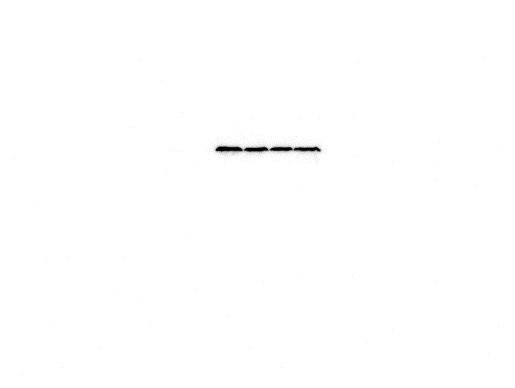
Fig. 1E: β-ACTIN Fig. 1E: IL4I1


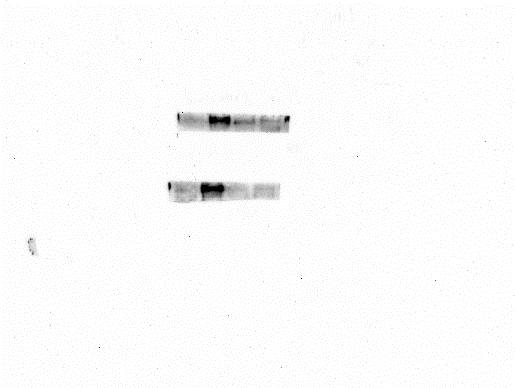


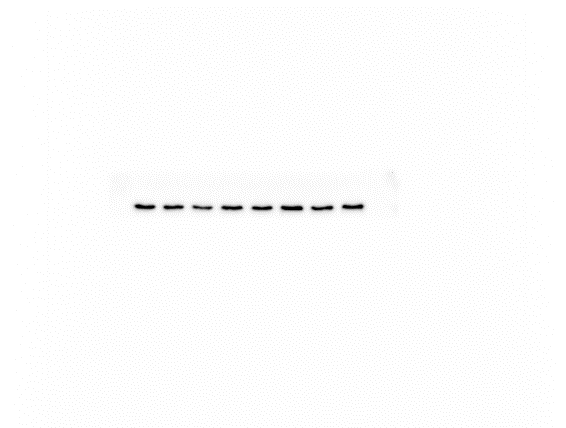

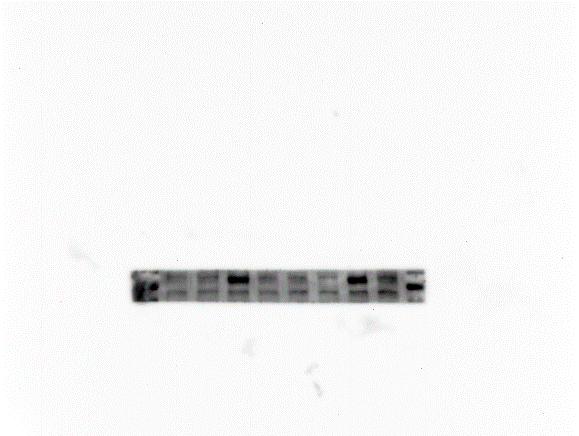
Fig. 1F, G: β-ACTIN Fig. 1F, G: IL4I1


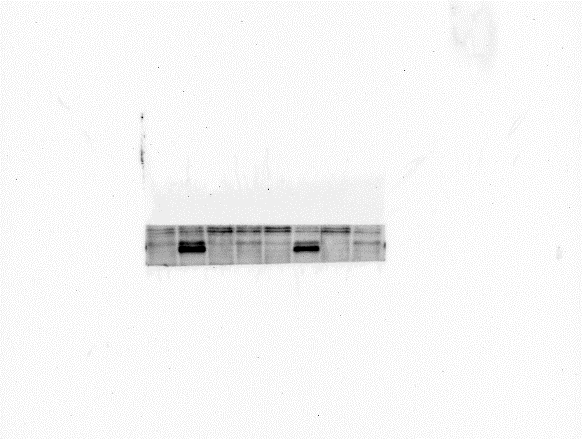

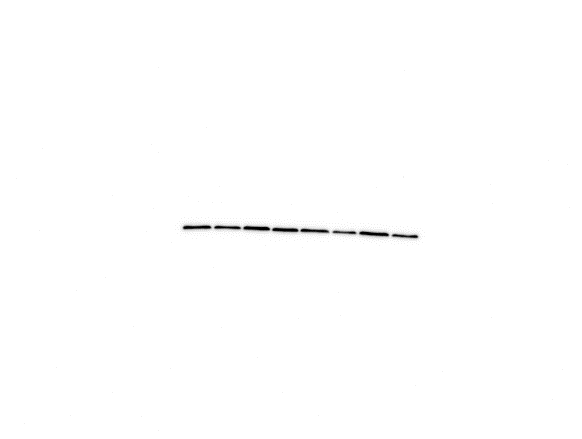
Fig. 2B: β-ACTIN Fig. 2B: IL4I1


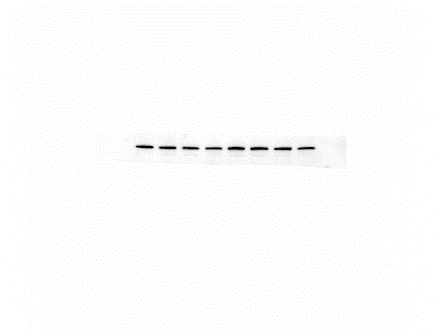
Fig. 5D: β-ACTIN Fig. 5D: LAMINB1


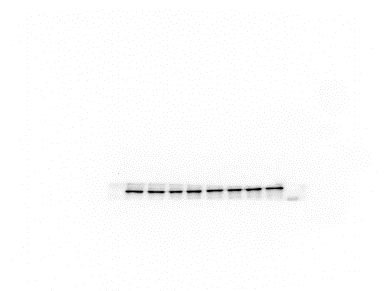


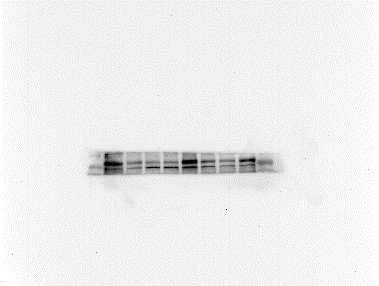
Fig. 5D: AHR in cytoplasm Fig. 5D: AHR in nucleus


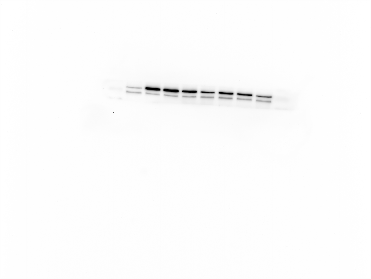


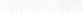

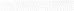

Supplement: Supplementary file 2 — Full length uncropped original western blots [file 41420_2023_1568_MOESM2_ESM.docx]
